# Involvement of TauT/SLC6A6 in Taurine Transport at the Blood–Testis Barrier

**DOI:** 10.3390/metabo12010066

**Published:** 2022-01-12

**Authors:** Yoshiyuki Kubo, Sakiko Ishizuka, Takeru Ito, Daisuke Yoneyama, Shin-ichi Akanuma, Ken-ichi Hosoya

**Affiliations:** 1 Department of Pharmaceutics, Graduate School of Medicine and Pharmaceutical Sciences, University of Toyama, 2630 Sugitani, Toyama 930-0194, Japan; s1860308@ems.u-toyama.ac.jp (S.I.); d2162302@ems.u-toyama.ac.jp (T.I.); m2061216@ems.u-toyama.ac.jp (D.Y.); akanumas@pha.u-toyama.ac.jp (S.A.); hosoyak@pha.u-toyama.ac.jp (K.H.); 2 Laboratory of Drug Disposition Pharmacokinetics, Faculty of Pharma-Sciences, Teikyo University, Kaga 2-11-1, Tokyo 173-8605, Japan

**Keywords:** taurine, transport, blood-testis barrier, seminiferous tubules, antioxidant, infertility

## Abstract

Taurine transport was investigated at the blood–testis barrier (BTB) formed by Sertoli cells. An integration plot analysis of mice showed the apparent influx permeability clearance of [^3^H]taurine (27.7 μL/(min·g testis)), which was much higher than that of a non-permeable paracellular marker, suggesting blood-to-testis transport of taurine, which may involve a facilitative taurine transport system at the BTB. A mouse Sertoli cell line, TM4 cells, showed temperature- and concentration-dependent [^3^H]taurine uptake with a K_m_ of 13.5 μM, suggesting that the influx transport of taurine at the BTB involves a carrier-mediated process. [^3^H]Taurine uptake by TM4 cells was significantly reduced by the substrates of taurine transporter (TauT/SLC6A6), such as β-alanine, hypotaurine, γ-aminobutyric acid (GABA), and guanidinoacetic acid (GAA), with no significant effect shown by L-alanine, probenecid, and L-leucine. In addition, the concentration-dependent inhibition of [^3^H]taurine uptake revealed an IC_50_ of 378 μM for GABA. Protein expression of TauT in the testis, seminiferous tubules, and TM4 cells was confirmed by Western blot analysis and immunohistochemistry by means of anti-TauT antibodies, and knockdown of TauT showed significantly decreased [^3^H]taurine uptake by TM4 cells. These results suggest the involvement of TauT in the transport of taurine at the BTB.

## 1. Introduction

Taurine (2-aminoethanesulfonic acid) is known as a β-amino acid that abundantly exists in the human body, and its involvement in various physiological events, such as neuroprotection and osmotic regulation, has been suggested by cumulative studies [1,2,3,4,5,6]. In the biosynthesis of taurine, cysteine sulfonate decarboxylase (CSD) is assumed to be a rate-limiting enzyme, the activity of which is relatively low in humans [7,8], and experiments in rodents have revealed that a dietary deficiency of taurine results in a dramatic decrease of taurine in the liver [9], suggesting the importance of the taurine transport system for regulating the concentration of taurine in organs and tissues [1,10,11].

Taurine is also abundant in human semen, the concentration of which (679 µM) is reported to be maintained at levels 10-fold higher than in the blood, suggesting the importance of taurine in the testis, including germ cells [12]. In particular, low expression of antioxidant enzymes such as superoxide dismutase (SOD) in the testis is known, and abnormalities in the motility and form of sperm are caused by oxidative stress [13]. The oral administration of taurine showed a protective effect against arsenic-induced oxidative stress in rats, supporting the significant role of taurine in the testis [14], and the administration of taurine in streptozotocin-induced diabetic rats suggested that taurine acts as an antioxidant in the seminiferous tubules harboring germ cells [15]. The study in diabetic rats also supported the contribution of taurine to the seminiferous tubules, since its dietary intake improved the sperm characteristics, including sperm count and motility, that are closely related to male infertility [16,17,18].

These pieces of evidence indicate the relevance of taurine to the healthy maintenance and pathogeneses of sperm, suggesting the importance of regulating the concentration of such antioxidants in the seminiferous tubules, where Sertoli cells form the blood–testis barrier (BTB) to separate germ cells from the circulating blood [19,20]. At the BTB, Sertoli cells are assumed to form tight junctions to suppress non-specific transport via the paracellular route [21] and have been reported to express various transporter molecules, such as glucose transporters (GLUTs/SLC2As), L-type amino acid transporter 1 (LAT1/SLC7A5), monocarboxylate transporter 8 (MCT8/SLC16A2), sodium-dependent vitamin C transporters (SVCTs/SLC23As), concentrative nucleoside transporters (SLC28As), equilibrative nucleoside transporters (SLC29As), P-glycoprotein (P-gp/MDR1/ABCB1), and multidrug resistance-associated protein 1 (MRP1/ABCC1), involved in the transport of nutrients, metabolites, and xenobiotics at the BTB [22,23,24,25,26,27], suggesting the involvement of certain transporters in regulating taurine concentration in the seminiferous tubules.

Molecular cloning has achieved the identification of transporter molecules, and mouse taurine transporter (TauT/SLC6A6) and mouse γ-aminobutyric acid (GABA) transporter 3 (GAT3/SLC6A13, the orthologue of rat GAT2) mediate Na^+^- and Cl^−^-dependent transport of taurine with K_m_ values of 4.50 and 540 µM, respectively [28,29,30]. In addition, H^+^-coupled amino acid transporter 1 (PAT1/SLC36A1) was reported to have a low affinity for taurine (K_m_ = 7.5 mM) [31]. In particular, a study on the blood-to-retina and blood-to-liver transport of taurine reported the major contribution of TauT and rat GAT2 to taurine transport at the inner blood–retina barrier (inner BRB) and the hepatocytes, respectively, revealing precise mechanisms for regulating taurine concentration in the body [10,11].

However, little is known about the transport of taurine at the BTB, and this mechanism was investigated in the present study. The blood-to-testis transport of [^3^H]taurine was investigated using integration plot analysis, and transport at the BTB was analyzed using a mouse-derived Sertoli cell line, TM4 cells [32]. In addition, the mRNA and protein expression of responsible transporters was analyzed using specific primers and antibodies.

## 2. Results

### 2.1. Integration Plot of [^3^H]taurine in Mice

**Figure 1:**
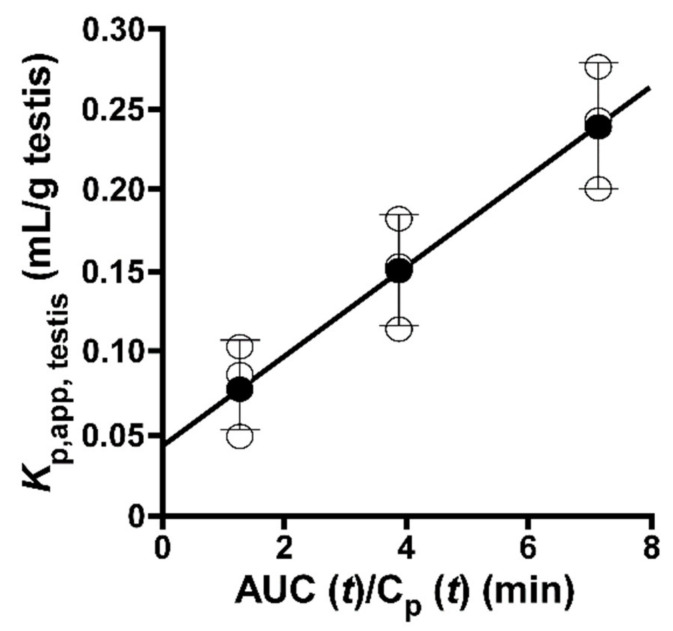
Blood-to-testis transport of [^3^H]taurine. Initial uptake of [^3^H]taurine by mouse testis. In integration plot analysis, [^3^H]taurine (0.33 µM, 2 µCi/mouse) was injected into the left common carotid vein. Clearance was obtained from the regression line slope (shown as a solid line). The solid line was fitted using a nonlinear least-squares regression analysis program (MULTI). Each open circle represents an individual data point, and each closed circle represents mean ± standard deviation (SD) (*n* = 3).

An integration plot analysis was carried out to evaluate the apparent influx clearance from circulating blood to the testis (CL_in, testis_) (Figure 1), and the results obtained show that the CL_in, testis_ of [^3^H]taurine was 27.7 ± 0.2 μL/(min·g testis), which is approximately 3-fold greater than the CL_in, testis_ of [^14^C]D-mannitol (8.79 ± 3.58 µL/(min·g testis)).

### 2.2. Uptake of [^3^H]taurine by TM4 Cells

**Figure 2:**
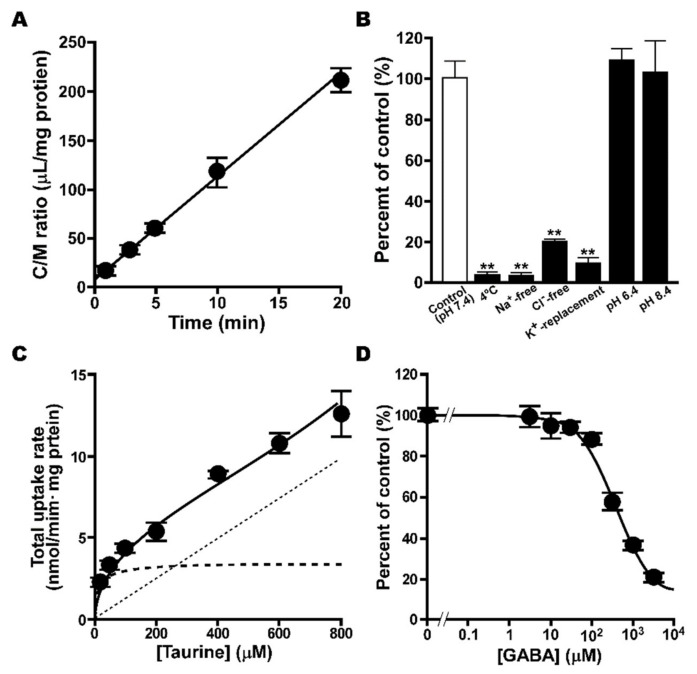
Uptake of [^3^H]taurine by TM4 cells. (**A**) Time course of [^3^H]taurine (16.5 nM, 0.1 µCi/well) uptake by TM4 cells was examined at 37 °C. (**B**) Effect of Na^+^, Cl^−^, membrane potential and extracellular pH on uptake was examined. Temperature dependence of uptake was examined at 4 °C for 5 min. (**C**) Concentration dependence of [^3^H]taurine uptake was examined over a concentration range of 20-800 µM. Dashed, dotted, and solid lines represent saturable, non-saturable, and overall uptake of taurine, respectively. (**D**) Inhibitory effect of GABA on uptake was examined in the absence or presence of unlabeled GABA at designated concentrations. Inhibitory effect of GABA was evaluated as IC_50_ value (378 µM). Unless otherwise noted, uptake was examined at 37 °C for 5 min. Each point or column represents mean ± SD (*n* = 3). ** *p* 0.01, significantly different from control.

An uptake analysis of [^3^H]taurine was carried out in TM4 cells, where a time-dependent increase was shown in [^3^H]taurine uptake for at least 20 min, with an initial uptake rate of 10.7 ± 0.2 μL/(min·mg protein) (Figure 2A). In addition, TM4 cells showed a significant reduction of [^3^H]taurine uptake at 4 °C, and the uptake was also significantly decreased in the assay with Na^+^-free, Cl^−^-free, and K^+^-replacement buffers, with no change shown by extracellular pH (Figure 2B). The uptake of [^3^H]taurine by TM4 cells took place in a concentration-dependent manner with K_m_ of 13.5 ± 3.8 μM, V_max_ of 3.42 ± 0.29 nmol/(min·mg protein), and K_d_ of 12.3 ± 0.65 μL/(min·mg protein), and the contribution ratio of the saturable process was calculated at approximately 95% (Figure 2C).

The inhibition of [^3^H]taurine uptake was also examined in TM4 cells, and uptake was significantly decreased in the presence of taurine, β-alanine, hypotaurine, GABA, and guanidinoacetic acid (GAA). No effect was observed in the presence of L-alanine, probenecid, and L-leucine (Table 1). In addition, the concentration-dependent inhibition of [^3^H]taurine uptake was examined for GABA, and its IC_50_ was calculated as 378 μM (Figure 2D).

### 2.3. Expression Analysis of TauT in TM4 Cells

**Figure 3:**
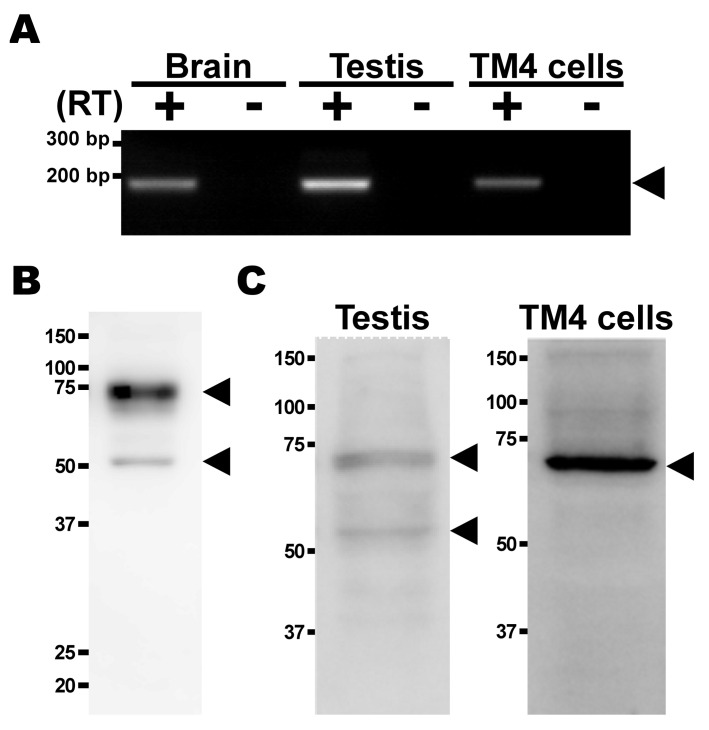
Expression study of TauT mRNA and protein. (**A**) mRNA expression of TauT in mouse brain, testis, and TM4 cells was analyzed by RT-PCR in the presence (+) or absence (−) of reverse transcriptase (RT). PCR products were analyzed by agarose gel electrophoresis and visualized by staining with ethidium bromide. Arrowheads indicate predicted product sizes. (**B**) Specificity and cross-reactivity of anti-TauT antibody were confirmed by Western blot analysis. Mouse TauT protein was detected at approximately 75 kDa (glycosylated) and 50 kDa (non-glycosylated) (arrowhead). (**C**) Protein expression of TauT in mouse testis and TM4 cells was analyzed by Western blot analysis with anti-TauT antibody. Mouse TauT protein at approximately 50 kDa and 70 kDa (arrowhead).

mRNA expression of TauT was examined, and agarose gel electrophoresis clearly detected the PCR product for mouse TauT (189 bp) in mouse brain used as a positive control (Figure 3A). The product was also detected in mouse testis and TM4 cells (Figure 3A). For the analysis of protein expression, anti-TauT polyclonal antibodies were prepared by immunizing female Hartley guinea pigs with the C-terminus peptide of rat TauT. The specificity and cross-reactivity of anti-TauT polyclonal antibodies were confirmed by Western blot analysis to specifically detect the signal of glycosylated (75 kDa) and non-glycosylated (50 kDa) TauT proteins in mouse kidney (Figure 3B), and another Western blot analysis with the antibodies clearly detected the signals for TauT protein in mouse testis and TM4 cells (Figure 3C).

**Figure 4:**
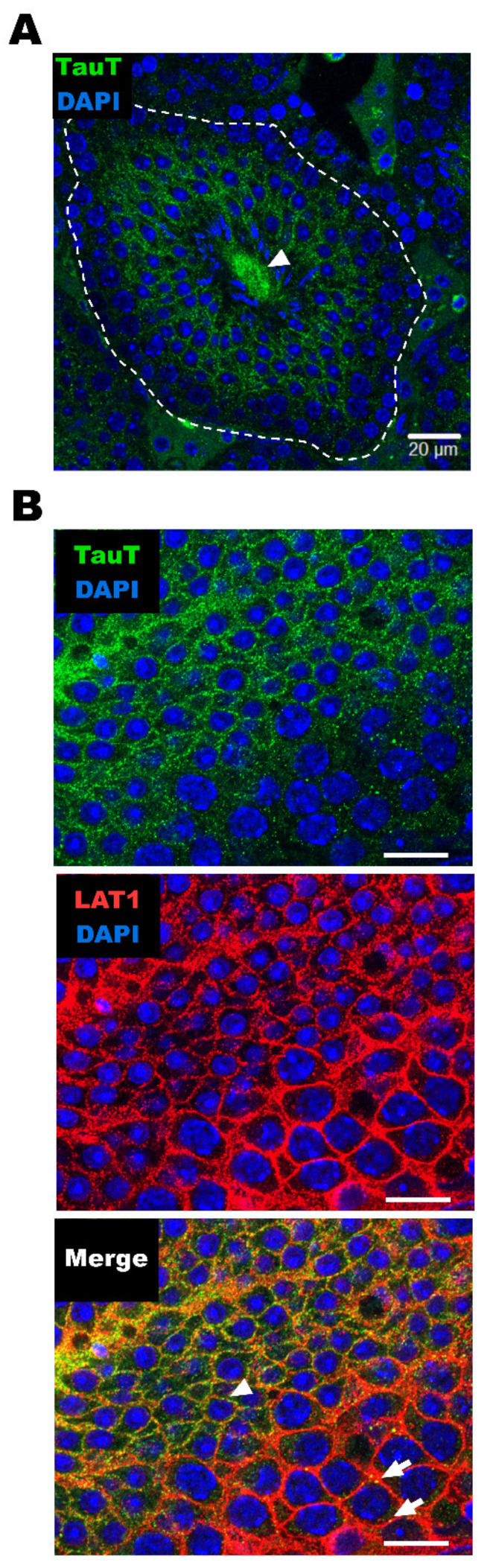
Immunohistochemistry of TauT in the mouse testis. (**A**) Mouse testis was analyzed with anti-TauT antibodies (green) and DNA intercalants DAPI (blue). TauT immunoreactivity was observed in seminiferous tubules (inside dotted line) and sperm (arrowhead). (**B**) Mouse seminiferous tubules were analyzed with anti-TauT antibody (green), anti-LAT1 antibody (red), and DNA intercalants DAPI (blue). Signal was observed on basal (arrow) and lumen (arrowheads) sides of seminiferous tubules. Scale bar: 20 µm.

Furthermore, the antibodies were recruited for the immunohistochemistry of TauT protein in mouse testis, and the fluorescence signal of TauT protein was detected in the seminiferous tubules (Figure 4A). In the study with anti-LAT1 antibodies, a strong signal merge of LAT1 and TauT was observed in the inner region of mouse seminiferous tubules during their weak merge in the outer region (Figure 4B) [26,30].

### 2.4. Knockdown of TauT in TM4 Cells

**Figure 5:**
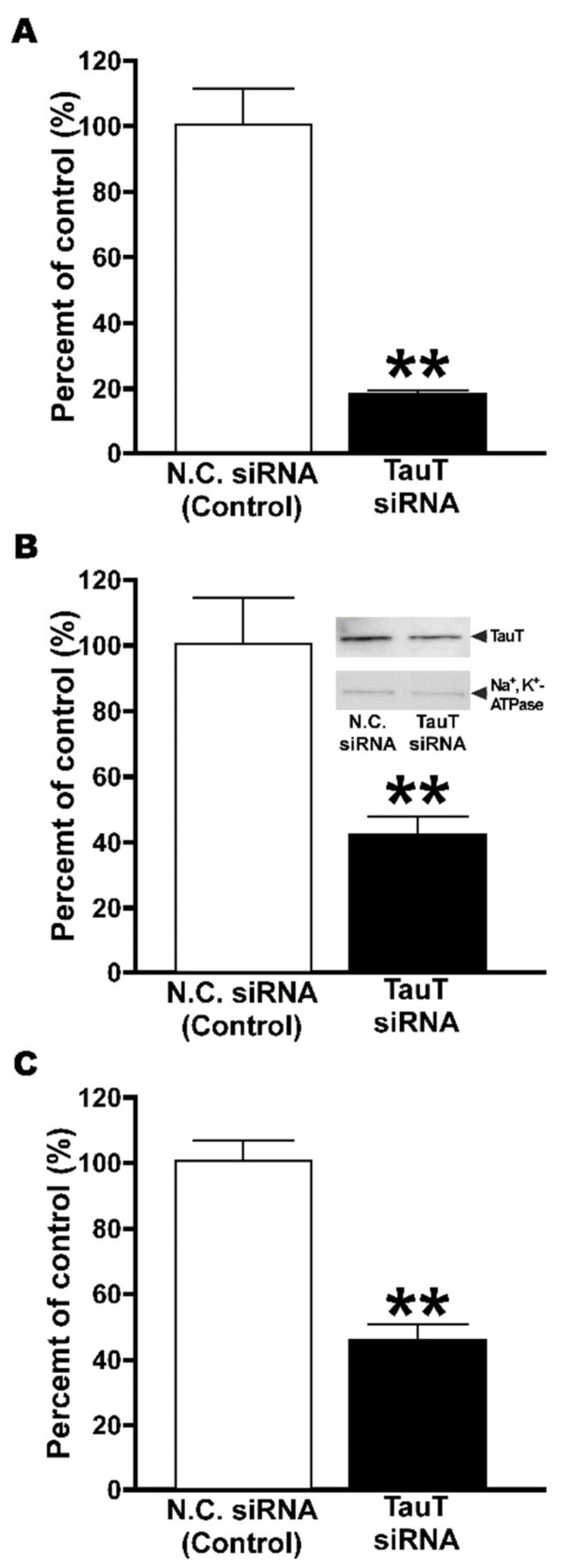
Knockdown analysis of TauT in TM4 cells. (**A**) Expression of TauT RNA was analyzed by quantitative real-time PCR and normalized with that of β-actin. (**B**) Expression of TauT protein was analyzed by Western blot with anti-TauT antibodies. Anti-Na^+^/K^+^-ATPase α1 antibodies were used to normalize the signal intensity of TauT (inset). (**C**) [^3^H]Taurine (16.5 nM, 0.1 µCi/well) uptake was performed at 37 °C for 5 min. TM4 cells were treated with negative control siRNA (N.C. siRNA) or TauT siRNA. Each column represents mean ± SD (*n* = 3). ** *p* 0.01, significantly different from control.

The effect of small interfering RNA specific for mouse TauT was examined in TM4 cells. Cells transfected with mouse TauT-specific siRNA showed a significant reduction of [^3^H]taurine uptake by 53% (Figure 5A), and also significantly exhibited reduced mouse TauT transcript and protein expression by 81 and 57%, respectively (Figure 5B,C).

## 3. Discussion

The abundance of taurine in human semen implicated the importance of taurine in the testis [12]. Studies on male infertility have reported the protective effect of taurine against oxidative stress, showing improvement in sperm characteristics, such as sperm count and motility [16,17,18], suggesting the significance of taurine regulation in the seminiferous tubules harboring germ cells. Therefore, the involvement of certain transport systems in taurine transport at the BTB formed by Sertoli cells can be assumed.

In the integration plot analysis, the CL_in,testis_ of [^3^H]taurine was 27.7 μL/(min·g testis), which was much higher than that of paracellular transport marker (Figure 1). This suggests the blood-to-testis transport of taurine, which may involve a facilitative transport system at the BTB.

The calculation of CL_in,testis_ for [^3^H]taurine implies the involvement of taurine transport at the BTB, and uptake analysis with TM4 cells, a mouse Sertoli cell line, suggests the involvement of a carrier-mediated process in the transport of taurine at the BTB, since the uptake of [^3^H]taurine took place in a time-, temperature-, and concentration-dependent manner (Figure 2). The estimation of kinetic parameters revealed a K_m_ value of 13.5 μM for taurine transport at the BTB, and further estimation of V_max_ (3.42 nmol/(min·mg protein)) and K_d_ (12.3 μL/(min·mg protein)) supported a substantial contribution of a saturable process, with a contribution ratio of 95%. In mice, taurine has been reported to be recognized by several transporters, such as TauT, mGAT3, and PAT1, and the K_m_ value (13.5 μM) obtained in TM4 cells was similar to that reported for mouse TauT (4.50 μM) [32]. In addition, the significant decrease in [^3^H]taurine uptake by TM4 cells was shown in Na^+^-free, Cl^−^-free, and K^+^-replacement buffers, while no significant change was exhibited by different extracellular pH levels (Figure 2). These results support the possible contribution of TauT and mGAT3 to the transport of taurine at the BTB, since they are known as Na^+^- and Cl^−^-dependent transporters during the pH-dependence of PAT1 [33].

In addition, the results obtained in the in vitro inhibition study support the possible contribution of TauT and mGAT3, since the uptake of [^3^H]taurine by TM4 cells was significantly reduced in the presence of their substrates alanine, β-hypotaurine, GABA, and GAA [1,28,34], while it was not changed in the presence of L-alanine, a substrate of PAT1 (Table 1) [33]. Furthermore, the study of concentration-dependent inhibition suggests a major contribution of TauT to taurine transport at the BTB, since the calculated IC_50_ for GABA (378 μM) in TM4 cells was very similar to that of TauT (501 μM) (Figure 2D) [35], whereas mGAT3 is reported to transport GABA and taurine with a K_m_ of 18.0 and 540 μM, respectively [30].

The expression analysis suggests that TauT has a certain role in the BTB, and RT-PCR and Western blot analysis clearly detected mouse TauT mRNA and protein in testis and TM4 cells (Figure 3), clearly suggesting the specificity and cross-reactivity of anti-TauT antibodies, the epitope of which is the C-terminus peptide of rat TauT. In the immunohistochemistry with the antibodies, during the difficulty of identifying the type of cells, the detected signals supported the expression of TauT protein in the seminiferous tubules at least, and the plasma membrane localization of TauT protein was also suggested in the seminiferous tubules, since the merging of LAT1 and TauT was observed (Figure 4).

Furthermore, knockdown analysis of TM4 cells clearly supports the major contribution of mTauT to taurine transport at the BTB, since the transfection of siRNA designed for mouse TauT showed significantly decreased taurine transport with significantly reduced expression of mouse TauT mRNA and protein, revealing a contribution ratio of 93% for TauT (Figure 5).

## 4. Materials and Methods

### 4.1. Reagents, Animals and Cells

Commercially available chemicals of reagent grade were used in the present study. [2-^3^H]Taurine ([^3^H]taurine, 30.0 Ci/mmol) and [1-^14^C]D-mannitol ([^14^C]D-mannitol, 58 mCi/mmol) were purchased from American Radiolabeled Chemicals (St. Louis, MO, USA). TM4 cells, a mouse-derived Sertoli cell line, were purchased from the UK Health Security Agency’s European Collection of Authenticated Cell Cultures (Salisbury, UK). Horse serum and fetal bovine serum for culturing TM4 cells were purchased from Thermo Fisher Scientific (Waltham, MA, USA) and SAFC biosciences (Lenexa, KS, USA), respectively. Male ddY mice (8 weeks old) and female Hartley guinea pigs were purchased from Japan SLC (Hamamatsu, Japan) and were used in accordance with the guidelines for animal experiments (University of Toyama; registration #A2020PHA-4 and -5).

### 4.2. Integration Plot Analysis

Integration plot analysis was used to evaluate the apparent influx clearance (CL_in, testis_) of [^3^H]taurine from the circulating blood to the testis [36]. Briefly, after anesthetizing mice with an intraperitoneal injection of pentobarbital (6.48 mg/kg), [^3^H]taurine (2 μCi/mouse) in 200 µL buffer (141 mM NaCl, 4.0 mM KCl, 10 mM 2-[4-(2-hydroxyethyl)piperazin-1-yl]ethanesulfonic acid (HEPES), and 2.8 mM CaCl_2_) was injected into the internal jugular vein. Blood samples were collected at designated times, and mice were decapitated to collect the testes, which were lysed in 2N NaOH. Plasma samples were prepared by centrifugation (5000× *g*, 4 °C, 10 min) of collected blood samples, and radioactivity in the blood, plasma, and testis samples was determined by means of a liquid scintillation counter (LSC-7400, Hitachi Healthcare Manufacturing, Chiba, Japan). In the present study, [^3^H]D-mannitol (1 µCi/mouse) was also tested as a non-permeable paracellular transport marker. CL_in,testis_ was calculated by Equation (1):

V_d_ = CL_in,testis_ × AUC(t)/C_p_(t) + V_i_ (1)

where the apparent volume of distribution to the testis, the area under the plasma concentration time curve of the compound from time 0 to t, the plasma concentration of the compound at time t, and the rapidly equilibrated distribution volume of the compound in the testis are expressed as V_d_ (µL/g testis), AUC(t) (dpm min/mL), C_p_(t) (dpm/mL), and V_i_ (µL/g testis), respectively.

### 4.3. Cell Uptake Analysis

TM4 cells were cultured on TrueLine cell culture dishes (NIPPON Genetics, Tokyo, Japan) in a humidified environment at 37 °C and 5% CO_2_. For culturing TM4 cells, Dulbecco’s modified Eagle medium (D-MEM)/Ham’s F-12 medium with L-glutamine (FUJIFILM Wako Pure Chemical Corporation, Osaka, Japan) was supplemented with 100 U/mL benzylpenicillin, 100 µg/mL streptomycin sulfate, 10% horse serum, and 5% fetal bovine serum [32]. The medium was changed every 3–4 days, and trypsin-EDTA was used for cell passage.

In the uptake study, TM4 cells (1.0 × 10^5^ cells/well) were seeded on Corning BioCoat poly-D-lysine-coated 24-well clear flat bottom TC-treated multiwell plates (Corning, Corning, NY, USA). After culturing for 2 days, the cells were rinsed 3 times with extracellular fluid (ECF) buffer (122 mM NaCl, 25 mM NaHCO_3_, 3 mM KCl, 1.2 mM MgSO_4_, 0.4 mM K_2_HPO_4_, 10 mM HEPES, 10 mM D-glucose, 1.4 mM CaCl_2_, pH 7.4) warmed to 37 °C, and incubated in 200 µL ECF buffer containing [^3^H]taurine (0.1 µCi/well) at 37 °C. As reported elsewhere [37], radioactivity measurements were performed using a liquid scintillation counter (LSC-7400, Hitachi Healthcare Manufacturing) after the cells were lysed with 1N NaOH and neutralized with 1N HCl. [^3^H]Taurine uptake by TM4 cells was expressed by the cell-to-medium (C/M) ratio (µL/mg protein) obtained in Equation (2), and cellular protein content was determined using a DC protein assay kit and microplate reader (Model 680) purchased from Bio-Rad (Hercules, CA, USA)

Cell-to-medium ratio = ([^3^H] dpm per cell protein (mg))/([^3^H] dpm per μL ECF-buffer) (2)

As described previously [36], in the study of concentration dependence, kinetic parameters were obtained by a nonlinear least-squares regression analysis program (MULTI) [38] and Equation (3):

V = V_max_ × C/(K_m_ + C) + K_d_ × C (3)

where C, V, K_m_, V_max_, and K_d_ are the substrate concentration, the uptake rate, the Michaelis constant, and the maximal uptake rate and non-saturable uptake clearance, respectively.

MULTI was also used to calculate the half maximal inhibitory concentration (IC_50_) by Equation (4) [37]:

P = (P_max_ − P_min_)/[(1 + (I/IC_50_)^n^] + P_min_ (4)

where P and P_max_ are the relative [^3^H]taurine uptake (%) in the presence or absence of γ-aminobutyric acid (GABA), respectively, and P_min_, n, and I are the inhibitor-insensitive component of relative [^3^H]taurine uptake with GABA, the Hill coefficient, and the concentration of GABA, respectively.

### 4.4. mRNA Expression Analysis

Total RNA extraction and mRNA expression analysis using reverse transcription polymerase chain reaction (RT-PCR) were carried out as reported previously [39]. Briefly, the total RNA of TM4 cells was extracted using an RNeasy Micro Kit (Qiagen, Venlo, Netherlands) and ReverTraAce (TOYOBO, Osaka, Japan), and oligo dT primer was used for reverse transcription. Veriti Thermal Cycle (Thermo Fisher Scientific, Waltham, MA, USA) and ExTaq (Takara, Shiga, Japan) were used in the PCR for mouse TauT through 30 cycles at 94 °C for 30 s, 57 °C for 30 s, and 72 °C for 45 s. The nucleic acid alignment of primers for mouse TauT (NM_009320) was as follows: forward primer was 5′-GCGTTTCCCGTACCTCTGC-3′ and reverse primer was 5′-ATGGATGCGTAGCCAATGCC-3′. Amplified products in PCR were subjected to agarose gel electrophoresis, followed by ultraviolet visualization with ethidium bromide.

In quantitative real-time PCR, Mx3005P (Agilent Technologies, Santa Clara, CA, USA), SYBR Premix Ex Taq, and ROX reference dye (Takara, Shiga, Japan) were used through 35 cycles at 95 °C for 30 s, 57 °C for 30 s, and 72 °C for 30 s [39]. The nucleic acid alignment of primers was as follows: forward primer was 5′-GCGTTTCCCGTACCTCTGC-3′ and reverse primer was 5′-ATGGATGCGTAGCCAATGCC-3′ for mouse TauT (NM_009320), and forward primer was 5′-TCATGAAGTGTGACGTTGACATCCGT-3′ and reverse primer was 5′-CCTAGAAGCATTTGCGGTGCACGATG-3′ for β-actin (NM_031144.3). The initial amount of mouse TauT transcripts was estimated by determining the threshold cycle number, and a standard curve was prepared by the control plasmids harboring the gene fragment of mouse TauT.

### 4.5. Protein Expression Analysis

Anti-TauT polyclonal antibodies were prepared by using the epitope encompassing 30 amino acid residues (REGATPFHSRATLMNGALMKPSHVIVETMM) located at the C-terminus region of rat TauT (NP_058902). Male Hartley guinea pigs were immunized with the glutathione S-transferase (GST)-tagged epitope peptide, and the titer and specificity of antibodies were improved in affinity column purifications after blood serum was collected. Western blot analysis was performed as described elsewhere [36]. Briefly, the crude membrane fraction (20 μg protein) prepared from TM4 cells was analyzed, with anti-TauT polyclonal antibodies (1.0 μg/mL) and horseradish peroxidase-conjugated anti-guinea pig IgG antibodies (Merck Millipore, Burlington, MA, USA) used as primary and secondary antibodies, respectively. In the analysis of Na^+^/K^+^-ATPase α-1 subunit, a plasma membrane marker, anti-Na^+^/K^+^-ATPase α-1 antibodies (0.5 μg/mL; Merck Millipore, Burlington, MA, USA) and horseradish peroxidase-conjugated anti-mouse IgG antibodies (Merck Millipore) were used. ECL Prime Western Blotting Detection System (Merck Millipore, Burlington, MA, USA) and Luminescent Image Analyzer (LAS-4000, FUJIFILM) were used for signal detection.

Immunohistochemistry was performed as reported previously [36], and an LSM780 confocal microscope (Carl Zeiss, Oberkochen, Germany) was used. After tissue fixation with 4% paraformaldehyde, a cryostat (CM1900, Leica, Wetzlar, Germany) was used to prepare frozen sections (12 μm thickness) of ddY mice testes, which were mounted on glass slides for blocking with 10% goat serum (Nichirei, Tokyo, Japan) at room temperature. Anti-TauT polyclonal antibodies (2.0 μg/mL) and rabbit polyclonal anti-LAT1 antibodies (3.0 μg/mL; Trans Genic, Fukuoka, Japan) were applied as the primary antibodies. Alexa Fluor 488-conjugated goat anti-guinea pig IgG and CyTM3-conjugated donkey anti-rabbit IgG antibodies were obtained from Thermo Fisher Scientific and Jackson ImmunoResearch, respectively, and were used as secondary antibodies. After being treated with 4′,6-diamidino-2-phenylindole (DAPI) and VECTASHIELD mounting medium (Vector Laboratories, Burlingame, CA, USA), sections were examined by confocal microscopy.

### 4.6. Knockdown Analysis of TauT

Small interfering RNA (siRNA) of mouse TauT were designed with reference to previous reports. Stealth RNAi Negative Control Medium GC Duplexes were obtained from Thermo Fisher Scientific [39], and all processes of transfection to TM4 cells were carried out according to the manufacturer’s instructions [39]. In brief, siRNA (30 pmol/well) was introduced to TM4 cells cultured on 6-well plates by means of Lipofectamine RNAiMAX (5 μL/well; Thermo Fisher Scientific). The uptake study was initiated 48 h after transfection, and the knockdown efficiency was confirmed by Western blot analysis and quantitative real-time PCR.

### 4.7. Statistical Analysis

All data in this paper are expressed as mean ± SD. Statistically significant differences (*p*-Value 1%) between two or more than three groups were determined by unpaired two-tailed Student’s *t*-test or one-way ANOVA by Dunnett’s test, respectively.

## 5. Conclusions

Clinically, hormonal modulators and herbal medicines have been used in the treatment of male infertility [40]. It is also thought that taurine is promising in the treatment of male infertility, since it has a protective effect against oxidative stress in the testes to improve sperm characteristics [14,15,16,17,18] and has been assumed not to produce adverse effects [41]. In the present study, the involvement of a transport system in the blood-to-testis transport of taurine is suggested, implying a facilitative transport system for taurine at the BTB. Uptake and expression studies have suggested that TauT largely contributes to the influx transport of taurine at the BTB, and it is assumed that TauT has an important role in the protection of germ cells from oxidative stress by supplying taurine to seminiferous tubules. The present findings will be helpful for improving the treatment of male infertility in association with a better understanding of taurine regulation in the testis.

## Figures and Tables

**Table 1:** Effect of several compounds on [^3^H]taurine uptake by TM4 cells.

Compounds	Percentage of Control (%)		
Control	100	±	8
Taurine	3.70	±	0.26 **
β-Alanine	4.69	±	0.76 **
Hypotaurine	13.3	±	3.1 **
GABA	23.2	±	2.7 **
GAA	57.3	±	4.2 **
L-Alanine	82.1	±	2.0
Probenecid	98.7	±	3.9
L-Leucine	112	±	7

[^3^H]Taurine (16.5 nM, 0.1 µCi/well) uptake was performed at 37 °C for 5 min in the absence (control) or presence of test compounds (1 mM). Each value represents mean ± SD (*n* = 3–9). ** *p* 0.01, significantly different from control. GABA, γ-aminobutylic acid; GAA, guanidinoacetic acid.

## Data Availability

The data of this study are available from the corresponding author upon reasonable request.
